# Acid-Base and Plasma Biochemical Changes Using Crystalloid Fluids in Stranded Juvenile Loggerhead Sea Turtles (*Caretta caretta*)

**DOI:** 10.1371/journal.pone.0132217

**Published:** 2015-07-13

**Authors:** María Camacho, María del Pino Quintana, Pascual Calabuig, Octavio P. Luzardo, Luis D. Boada, Manuel Zumbado, Jorge Orós

**Affiliations:** 1 Veterinary Faculty, University of Las Palmas de Gran Canaria, Arucas (Las Palmas), Spain; 2 Department of Mathematics, University of Las Palmas de Gran Canaria, Las Palmas de Gran Canaria, Spain; 3 Tafira Wildlife Rehabilitation Center, Cabildo de Gran Canaria, Tafira Baja-Las Palmas de Gran Canaria, Spain; University of Bari, ITALY

## Abstract

**Aim:**

The aim of this study was to compare the efficacy and effects on acid-base and electrolyte status of several crystalloid fluids in 57 stranded juvenile loggerhead turtles.

**Methods:**

Within a rehabilitation program four different crystalloid fluids were administered (0.9% Na Cl solution; 5% dextrose + 0.9% Na Cl solutions 1:1; 0.9% Na Cl + lactated Ringer's solutions 1:1; lactated Ringer's solution). Crystalloid fluids were intracoelomically administered during three days (20 ml/kg/day). Animals were sampled at three different moments: Upon admission for evaluating the type of acid-base or biochemical disorder, post-fluid therapy treatment for controlling the evolution of the disorder, and post-recovery period for obtaining the baseline values for rehabilitated loggerhead turtles. Each sample was analyzed with a portable electronic blood analyzer for pH, pO_2_, pCO_2_, lactate, sodium, potassium, chloride, glucose, and BUN concentration. Admission and post-fluid therapy treatment values were compared with those obtained for each turtle immediately before release.

**Results:**

The highest percentage of acid-base recovery and electrolyte balance was observed in turtles treated with mixed saline-lactated Ringer’s solution (63.6%), followed by turtles treated with physiological saline solution (55%), lactated Ringer’s solution (33.3%), and dextrose-saline solutions (10%). Most turtles treated with lactated Ringer’s solution had lower lactate concentrations compared with their initial values; however, 66.6% of turtles treated with lactated Ringer’s solution had metabolic alkalosis after therapy. Significant higher concentrations of glucose were detected after saline-dextrose administration compared with all the remaining fluids.

**Conclusions:**

This is the first study evaluating the effects of several crystalloid fluids on the acid-base status and plasma biochemical values in stranded loggerhead sea turtles. Reference convalescent venous blood gas, acid-base, and plasma biochemical values, useful for veterinary surgeons involved in sea turtle conservation, are also provided.

## Introduction

Seven species of sea turtles are currently recognized, all of which are included on the Red List of the World Conservation Union [1]. The most common species around the Canary Islands is the loggerhead turtle (*Caretta caretta*), mainly coming from the US western Atlantic by the Gulf Stream [2].

Many veterinary surgeons are currently involved in sea turtle conservation in wildlife rehabilitation hospitals around the world. Clinical and pathological studies contribute to a better understanding of problems in stranded sea turtles and provide a basis to guide conservation efforts [3]. Dehydration, disturbances of acid-base homeostasis, and electrolyte imbalances are common in stranded sea turtles [3–7]. Variations of venous blood gas, acid-base, plasma biochemical and hematologic parameters of stranded loggerhead turtles according to the cause of stranding or disease have been reported [3,8]. However, no specific studies of fluid therapy in sea turtles have been reported.

Fluid therapy is an integral part of reestablishing and maintaining cellular homeostasis and although the principles of fluid therapy are universal across species lines, choosing the type of fluids to administer to a reptile is somewhat controversial [9]. Fluid choice should be based on patient assessment and plasma biochemistry and blood gas values [10]. However, although hematologic and plasma biochemical values from loggerhead turtles have been described [8, 11–13], reports of blood gas and acid-base status of loggerhead turtles are limited [3,4,14,15]. In addition, the wide range for some of these reference values usually makes difficult to assess the dehydration status and to choose the appropriate fluid.

The aim of this study was to compare the efficacy and effects on acid-base and electrolyte status of several crystalloid fluids in stranded juvenile loggerhead turtles.

## Material and Methods

### Ethics Statement

Sea turtle rehabilitation program at the TWRC was conducted with authorization of the Wildlife Department of the Canary Islands Government (Ms. Guacimara Medina), and the Environment Department of the Cabildo de Gran Canaria (Ms. María del Mar Arévalo). Animal work and all sampling procedures were specifically approved by the TWRC Animal Care Committee and the insular government Cabildo de Gran Canaria, and were consistent with standard vertebrate protocols and veterinary practices.

### Animals

A total of 57 loggerhead turtles admitted at the TWRC were included in this study. Details of the medical management and care of sea turtles at the TWRC have been previously reported [3,11]. All turtles selected for this study were identified as juvenile specimens on the basis of straight carapace length (SCL) [16,17]. On the day of admission, data collected included weight, SCL and primary cause of stranding. Visualization of the gonads via surgery or endoscopy was not performed. The median and standard deviation of SCL and weight of juvenile turtles were 29.83 ± 6.97 cm (range: 18–46 cm) and 4.77 ± 3.14 kg (range: 1.03–14.5 kg), respectively. The causes of stranding were entanglement in fishing nets (n = 48; 84.2%), malnutrition (n = 2; 3.5%), traumatic injuries caused by boat strikes (n = 1; 1.8%), buoyancy disorders (n = 1; 1.8%), plastic ingestion (n = 1; 1.8%) and unidentified causes (n = 4; 7%).

### Methodology

Four different groups were established according to the different crystalloid fluids administered. Group 1 (n = 12): 0.9% Na Cl solution; group 2 (n = 12): 5% dextrose + 0.9% Na Cl solutions 1:1; group 3 (n = 12): 0.9% Na Cl and lactated Ringer's solutions 1:1; group 4 (n = 11): lactated Ringer's solution. We also included an untreated control group (n = 10). Crystalloid fluids were administered during three days consecutively (20 ml/kg/day) utilizing the commonly used in sea turtles intracoelomic (IC) route via the inguinal fossa [18–20]. The fluid therapy treatment started on day of admission and, when necessary, other medical and surgical procedures were done (e.g. amputation of the injured flipper, proper cleaning and debriding of the external traumatic injuries, antimicrobial treatment, etc). Sea turtles were placed for rehabilitation individually in outdoor pools with continuous flow of sea water, capacity of 10,000 l, and depth of 1 m, providing plenty of room for swimming. Clinical evaluation, including physical examination, evaluation of swimming activity, core body temperature (measured from the cloaca), food ingestion, and weight was performed daily following a complete clinical assessment protocol [12,19,21].

In order to evaluate the metabolic and respiratory status of stranded loggerhead turtles, all the animals were sampled at three different moments: upon admission (pre-treatment) for evaluating the type of acid-base or biochemical disorder, post-fluid therapy treatment for controlling the evolution of the disorder, and post-recovery period for obtaining the baseline values for rehabilitated loggerhead turtles. Same time intervals were used for collecting blood samples from the control turtles. Admission and post-fluid therapy treatment values were compared with those obtained for each turtle immediately before release. Each turtle was released when it was determined to be convalescent on the basis of clinical parameters, and the turtle was in good physical condition.

To minimize artifactual changes in blood parameters due to animal handling, 1 ml of venous blood was anaerobically collected from the cervical sinus of each turtle into a nonheparinized syringe immediately at time of admission. Each sample was immediately analyzed with a portable electronic blood analyzer (i-STAT, Heska, Loveland, CO) for pH, pO_2_, pCO_2_, and lactate concentration with CG4+ cartridges (Heska, Loveland, CO) for those analytes and for concentrations of sodium, potassium, chloride, glucose, and BUN with EC8+ cartridges (Heska, Loveland, CO) for those analytes. Because the portable electronic blood analyzer performs analysis of samples at 37°C, the temperature of each turtle was taken to correct the parameters with equations and values considered more appropriate for sea turtles than the portable electronic blood analyzer human-derived algorithms [22–24]. The body temperature of each turtle was recorded with a digital thermometer (Digi-Sense Thermocouple T, Cole-Parmer Instrument Co, Vernon Hills, IL) inserted at least 10 cm into the cloaca. The pH, pO_2_, and pCO_2_ were corrected for the turtle’s body temperature via the following equations:

Temperature corrected pH=pH+0.014 (ΔT)

$${\text{Temperature corrected pCO}}_{2}={\text{pCO}}_{2}\;\left(10^{-0.019\Delta \text{T}}\right)$$

$${\text{Temperature corrected pO}}_{2}={\text{pO}}_{2\;}\left(10^{-0.0058\Delta \text{T}}\right)$$

where ΔT = 37°C−body temperature

The temperature corrected HCO_3_^−^ concentration was calculated via the Henderson-Hesselbach equation. The solubility coefficient for CO_2_ (αCO_2_) and pK were calculated for each patient via species-specific equations for sea turtles [15,24].

Anion gap (mmol/l) and osmolality (mOsm/kg) were calculated by the following formulas:

$$\text{Anion gap}=\left(\text{sodium concentration}+\text{potassium concentration}\right)-\left(\text{chloride concentration}+{\text{temperature corrected HCO}}_{3}^{-}\text{ concentration}\right)$$

Osmolality = 2 (sodium concentration + potassium concentration) + (glucose concentration⁄18) + (BUN concentration/2.8) [26]

### Statistical analysis

Statistical analysis was performed using the SPSS statistical package v.21.0 (SPSS Inc., Chicago IL). Analysis of variance (ANOVA) for repeated measures was used to evaluate the changes in the parameters over the course of the study and between-groups comparison. The Bonferroni test was used to adjust multiple comparisons. Data non normally distributed were analyzed using Kruskas-Wallis test to compare among groups differences. Values of *P* < 0.05 were considered significant

## Results

**Fig 1:**
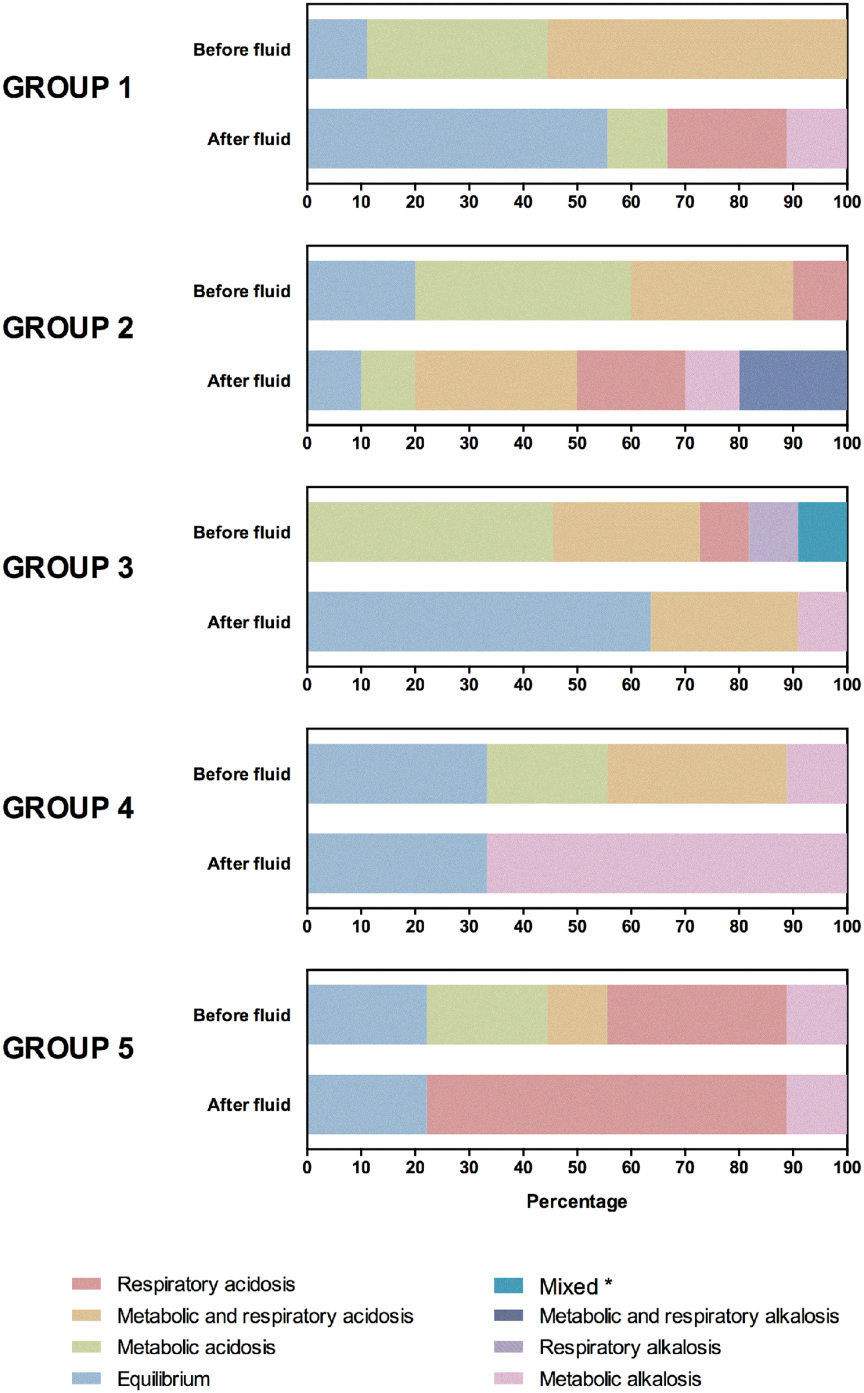
Acid-base status upon admission and after fluid therapy for each group of loggerhead sea turtles. Group 1: 0.9% Na Cl solution; group 2: 5% dextrose + 0.9% Na Cl solutions 1:1; group 3: 0.9% Na Cl and lactated Ringer's solutions 1:1; group 4: lactated Ringer's solution; group 5: untreated turtles. *metabolic acidosis and respiratory alkalosis.

Forty turtles (70.2%) had some type of acid-base disorder at the time of admission. Of these, 32 (80%) had metabolic acidosis. In most cases metabolic acidosis was associated with an elevation of anion gap (n = 23). However, hyperchloremia was observed in 4 turtles. The remaining turtles (n = 5) did not show any alteration of anion gap or chloride. The highest percentage of acid-base recovery and electrolyte balance after three days of therapy was observed in the group 3 (physiological saline + lactated Ringer’s solution) (63.6%), followed by turtles treated with physiological saline solution (55%), lactated Ringer’s solution (33.3%), and dextrose-saline solutions (10%). Two turtles (22.2%) belonging to the control group showed acid-base recovery after three days of therapy (Fig 1).

Values and descriptive statistics for acid-base and biochemical parameters at the time of admission, after fluid therapy and before release for treated groups and control group are presented in Tables 1 and 2, respectively. In addition, convalescent values of all the turtles included in this study are given in Table 3.

**Table 1:** Mean and standard deviation of pH, blood gas and plasma biochemical values during times of sampling of the different treatment groups.

*Parameters*	Group 1			Group 2			Group 3			Group 4		
	Upon admission	After fluid	Previous to release	Upon admission	After fluid	Previous to release	Upon admission	After fluid	Previous to release	Upon admission	After fluid	Previous to release
**pH**	7.50 ± 0.1	7.56 ± 0.06	7.58 ± 0.08^c^	7.53 ± 0.06	7.53 ± 0.07	7.58 ± 0.05^c^	7.58 ± 0.06	7.57 ± 0.06	7.61 ± 0.05	7.51 ± 0.07^a^	7.6 ± 0.06	7.55 ± 0.03
**pCO_2_ (Torr)**	30.08 ± 3.40^a^	36 ± 5.40	33 ± 5.70	33.63 ± 4.90	40 ± 7.80	34 ± 4.30	26.5 ± 3.70^a^	34.2 ± 5.90	33 ± 6.90	29.2 ± 3.60^a^	34.5 ± 6.30	32 ± 4.20
**pO_2_ (Torr)**	76 ± 10.30	62.6 ± 12.30	65 ± 22.10	70.5 ± 10.80	64.5 ± 11.8	60 ± 10.80	72.8 ± 11.1^a^	58.3 ± 7.80	61.6 ± 9.0	67 ± 9.80	64 ± 5.30	65 ± 9.20
**HCO_3_ (mmol/l)**	29 ± 6.20^a^	39.2 ± 5.60	38.6 ± 5.70^c^	33.4 ± 6.20^a^	40.3 ± 4.80	49.7 ± 4.80^c^	32.8 ± 6.10^a^	40.2 ± 7.80	42 ± 6.40^c^	27.9 ±5.50^a^	41.3 ± 4.90	34.6 ± 6.10^c^
**Lactate (mmol/l)**	2.01 ± 1.69	0.99 ± 0.60	0.73 ± 0.40	1.96 ± 1.54	1.30 ± 1.20	0.44 ± 0.30^c^	1.41 ± 1.03	1.17 ± 1.53	0.73 ± 0.54	2.90 ± 2.02	1.05 ± 0.80	0.53 ± 0.30^c^
**Sodium (mmol/l)**	148 ± 3.04	145.7 ± 2.3^b^	150 ± 5.06	148.5 ±1.90^a^	146.2 ± 2.9	148.9 ± 3.50	148.7 ± 2.5^a^	145.8 ± 2.8	148.2 ± 2.1	147 ± 2.50^a^	144.6 ± 2.7^b^	149.1 ± 1.90
**Potassium (mmol/l)**	3.5 ± 0.60^a^	2.80 ± 0.30	3.30 ± 0.40	3.40 ± 0.40^a^	2.70 ± 0.30	3.10 ± 0.40	3.30 ± 0.50	2.60 ± 0.50	3 ± 0.40	3.30 ± 0.40^a^	2.90 ± 0.30	3.20 ± 0.50
**Chloride (mmol/l)**	113.30 ± 4^a^	106.2 ±3.70	110.2 ± 3.80	111.3 ± 3.4^a^	105.8 ± 4.02	109.3 ± 4.3	111 ± 3.90^a^	104.3 ± 6.4	106.5 ± 4.4^c^	113.4 ± 3.5^a^	105 ± 3.80	110.7 ± 5.80
**Agap (mmol/l)**	9.80 ± 4.80^a^	3 ± 4.30	4.50 ± 7.50	8.10 ± 3.50^a^	2.80 ± 4.20	3 ± 5.90^c^	8.20 ± 3.90^a^	3.80 ± 4.40	2.70 ± 3.1^c^	9 ± 3.20^a^	1.30 ± 4.40^b^	6.10 ± 3.10
**Glucose (mg/dl)**	108 ± 26.60	119.2 ± 17.2	117.4 ± 22.2	111 ± 77.5^a^ ^,^ ^e^	207 ± 61^b^	109.4 ± 15.2	106 ± 32.40	108 ± 28.60	111 ± 12.6	115.2 ± 22.3	103.2 ± 19.2	107 ± 16.50
**BUN (mg/dl)** ^d^	58.3 ± 25.60^a^	88.5 ± 31.2^b^	136.5 ± 11^c^	71.2 ± 25.40^a^	84 ± 30.1^b^	134 ± 10.8^c^	71.2 ± 25.30^a^	86.2 ± 36.6^b^	125 ± 25.2^c^	57 ± 21.80^a^	81.4 ± 30.4^b^	135 ± 13^c^
**Osmolality (mOsmol/kg)** ^d^	331 ± 11.70	335 ± 13^b^	362 ± 11^c^	337 ± 13.70	341 ± 13^b^	358 ± 8.70^c^	335.5 ± 12.70	334 ± 17^b^	353.3 ± 8.7^c^	327.5 ± 11.3	330 ± 11.7^b^	358 ± 7.30^c^

Group 1: 0.9% Na Cl solution; Group 2: Dextrose 5% + 0.9% Na Cl solution (1:1); Group 3: 0.9% Na Cl + lactated Ringer’s solution (1:1); Group 4: lactated Ringer’s solution.

^a^Statistically significant differences between initial and after therapy values;

^b^statistically significant differences between after therapy and convalescent values;

^c^statistically significant differences between initial and convalescent values;

^d^mean and standard deviation convalescent BUN and Osmolality underestimate the true values because BUN values exceeded the analytic range of the analyzer (140 mg/dl);

^e^one turtle had glucose levels < 20 mg/dl.

**Table 2:** Mean and standard deviation of pH, blood gas and plasma biochemical values during times of sampling of untreated turtles (group 5).

Parameters	Upon admission	Control day	Previous to release
**pH**	7.52 ± 0.10	7.51 ± 0.08	7.57 ± 0.03
**pCO_2_ (Torr)**	35.20 ± 5.03	45 ± 8.60^b^	35.50 ± 5.30
**pO_2_ (Torr)**	67.10 ± 13	59 ± 9.20	59 ± 10.20
**HCO_3_ (mmol/l)**	34.70 ± 6.60^a^	43.60 ± 7.40	40.70 ± 6.70^c^
**Lactate (mmol/l)**	1.11 ± 0.70	0.75 ± 0.60	0.42 ± 0.20
**Sodium (mmol/l)**	146.30 ± 4.30	146.10 ± 2.50	148.80 ± 4.60
**Potassium (mmol/l)**	3.60 ± 0.33^a^	2.90 ± 0.45	3.30 ± 0.60
**Chloride (mmol/l)**	112.60 ± 3.40^a^	106.30 ± 4.30	107.20 ± 5.10^c^
**Agap (mmol/l)**	2.55 ± 7.64	(-0.9) ± 6.50	4.20 ± 6.90
**Glucose (mg/dl)**	115.9 ± 33.40	106.80 ± 21	114.8 ± 12.60
**BUN (mg/dl)** ^**d**^	71.70 ± 28.20^a^	98.70 ± 28.20^b^	140 ± 0.00^c^
**Osmolality (mOsmol/kg)** ^**d**^	330.80 ± 12.60^a^	339.4 ± 12.50^b^	360.6 ± 9.10^c^

^a^Statistically significant differences between initial and control day values;

^b^statistically significant differences between control day and convalescent values;

^c^statistically significant differences between initial and convalescent values;

^d^mean and standard deviation convalescent BUN and Osmolality underestimate the true values because BUN values exceeded the analytic range of the analyzer (140 mg/dl).

**Table 3:** Mean and standard deviation of convalescent values and statistical comparison with admission and post fluid therapy values.

Parameters	Convalescent values			
	Mean (median) ± SD	10-90th percentile	*P* ^a^	*P* ^b^
**pH**	7.58 (7.58) ± 0.05	7.52–7.63	< 0.001	ns
**pCO_2_ (Torr)**	33.49 (33.27) ± 5.30	27.21–41.30	< 0.05	< 0.001
**pO_2_ (Torr)**	64.05 (60.16) ± 19.80	48.87–78.16	< 0.001	ns
**HCO_3_ (mmol/l)**	39.15 (38.23) ± 6.26	32.60–47.35	< 0.001	ns
**Lactate (mmol/l)**	0.57 (0.39) ± 0.38	0.30–1.10	< 0.001	< 0.01
**Sodium (mmol/l)**	148.90 (149) ± 3.70	145–153	ns	< 0.001
**Potassium (mmol/l)**	3.20 (3.10) ± 0.46	2.60–4	< 0.01	< 0.001
**Chloride (mmol/l)**	108.80 (109) ± 4.84	112.80–114.20	< 0.001	< 0.001
**Agap (mmol/l)**	4 (5) ± 5.54	(-4.11)-11.20	< 0.001	< 0.05
**Glucose (mg/dl)**	112 (110) ± 16.20	95.60–131.20	ns	ns
**BUN (mg/dl)^c^**	133.90 (140) ± 15.07	112.80–140	< 0.001	< 0.001
**Osmolality (mOsmol/kg)^c^**	358.25 (358.62) ± 9.26	344.83–369.10	< 0.001	< 0.001

*P* ^*a*^ Statistical comparison between convalescent and admission values.

*P* ^*b*^ Statistical comparison between convalescent and post fluid therapy values.

^c^Mean and standard deviation convalescent BUN and Osmolality underestimate the true values because BUN values exceeded the analytic range of the analyzer (140 mg/dl).

ns: no significance

Several statistically significant differences were observed between initial parameters and those values obtained after therapy and convalescent values. Mean post-therapy pH levels were significantly higher after lactated Ringer’s solution administration. Mean initial pCO_2_ values were significantly lower than post-therapy values in groups 1, 3 and 4. Compared with initial values, bicarbonate values were higher after fluid therapy in all groups. Although no significant differences were observed among lactate values, we detected a decrease of lactate concentration during times of sampling in all groups. Significant lower values of sodium were detected in groups 1 and 4 after therapy compared with convalescent values. Potassium and chloride values were lower after therapy in all the groups compared with initial values. Furthermore, anion gap values decreased after fluid therapy. However, mean glucose values were significantly higher after dextrose-saline administration. BUN and osmolality values also increased in all groups compared with initial values.

On the other hand, when we analysed the potential differences between groups, only differences were observed for glucose and pCO_2_. Mean pCO_2_ was significantly higher in untreated turtles than in groups 1, 3 and 4. In addition, we detected significant higher concentrations of glucose after saline-dextrose administration compared with all the remaining therapy fluids (*P* < 0.01). Hyperglycemia was observed in the 91.7% (n = 11) of turtles after administered saline-dextrose fluid (median = 215 mg/dl; total range = 102–326 mg/dl). It is remarkable that 5 turtles included in this therapy group had hypoglycemia at the time of admission. Four of them had an abnormal elevation of glucose values after fluid therapy (including one turtle with an initial glucose value < 20 mg/dl). Moreover, turtles with normal (n = 4) or elevated (n = 3) initial glucose values also suffered hyperglycemia after therapy with salin-dextrose fluid.

## Discussion

This is the first study in which the acid-base and plasma biochemical changes after using several crystalloid fluids in stranded sea turtles have been evaluated. Fluids commonly used in chelonians include crystalloid fluids [19]. In this study only isotonic and hypotonic commercial preparations were administered, and no hypertonic crystalloid or colloid fluids were used. Crystalloids are the mainstay of the rehydration and maintenance phases of fluid therapy.

The highest percentage of acid-base recovery after three days of fluid therapy was observed in the group treated with mixed saline-lactated Ringer’s solution. Acidosis was the most frequent disorder in juvenile turtles upon admission at the TWRC. Metabolic acidosis is the most common disorder of acid-base balance encountered in sea turtle clinical practice and it is associated with an increased production of acid or loss of base [3]. In this study, low initial bicarbonate values associated with an elevation of anion gap and therefore increased lactic acid and decreased pCO_2_ values were presented in most of the turtles indicating metabolic acidosis. Although hyperchloremia was also observed in some turtles, this alteration was not common. The choice of fluid should depend on the type of clinical disorder and the associated fluid and electrolyte loss. Crystalloid fluids containing acetate, gluconate and lactate (ie, Plasmalyte-A, Normosol-R, lactated Ringer’s solution) could be used in reptiles [10]. These fluids are considered adequate for increasing the alkalinity and so counteract the acidosis [27]. Our results indicate that the buffered fluid (lactated Ringer’s solution) mixed with physiological saline could be indicated for restoring this acid-base disorder. The higher pCO_2_ values detected in untreated turtles compared with the other treated groups demonstrated a no solved respiratory acidosis disorder in this control group.

The use of lactated Ringer’s solution is controversial in reptiles. Some authors suggest that the lactate buffered fluids may not be appropriate for reptiles, including chelonians, because may exacerbate hyperlactatemia [10,18,28]. In addition, sea turtles can generate high concentrations of lactate after strong activity or forced submergence [4,29,30]. Because the rise of lactate concentration may produce acidosis, it has been suggested the use of non-lactate solutions, such as Plasmalyte-A or Normosol-R [28]. However, other authors stated that lactate solutions do not exacerbate acidosis in reptiles with appropriate liver function [10,19,31,32]. Thus, based on the common finding of acidosis in stranded loggerhead turtles [3], in cold-stunned Kemp’s ridley turtles [5] and in the present study, we suggest that buffer solutions should help counteract this disturbance.

In our study most turtles treated with lactated Ringer’s solution had lower lactate concentrations compared with their initial values. However, 66.6% of turtles treated with lactated Ringer’s solution had metabolic alkalosis after therapy, with significant higher pH values after therapy compared with their initial values. This result could indicate that lactate concentration of lactated Ringer’s solution is greater than required for some turtles. Furthermore, only 33% of turtles from this group had an acid-base equilibrium after therapy. For this reason, we recommend the administration of the mixed lactated-saline solution instead lactated Ringer’s solution until the acid-base disorder is restored. Then, it is possible to continue with a maintenance crystalloid fluid (0.9% NaCl), as other authors used [20].

The higher glucose concentrations observed in the turtles treated with dextrose-saline fluid were unexpected, taking into account the low initial glucose values observed in almost 50% of the turtles of this group. In addition, treatment with dextrose-saline solution presented the worst results restoring the acid-base balance. However, pancreatic and hepatic panels were not performed in our study. Hyperglycemia is commonly detected in sea turtles [3,5], being associated with stress in some cases [33,34], and of unknown origin in debilitated loggerhead turtles [35]. Hyperglycemia has also been reported after exogenous dextrose administration in reptiles [36]. Other causes for hyperglycemia in reptiles include liver disease, pancreatic disease, and overcompensation of gluconeogenic mechanisms [36]. Blood glucose determination is essential for choosing the appropriate fluid therapy. Dextrose solution is indicated in hypoglycemic patients. Glucose values should be determined until the condition is normalized and then continue with a maintenance crystalloid fluid [20]. However, further investigation of the pathophysiologic mechanism of hyperglycemia in sea turtles is necessary.

The increase of pH, bicarbonate and pCO_2_ values linked to the decrease of anion gap and lactate concentrations in groups 1, 3 and 4 showed the alkalization of acid-base status after therapy. These observations have been previously detected in sea turtles after a period of rehabilitation [3,5]. In addition, in a previous study, juvenile loggerhead turtles had lower initial levels of sodium and higher initial levels of chloride upon admission compared with their respective values obtained when turtles were fully recovered [3]. In the present study, lower concentrations of sodium, potassium and chloride after fluid therapy were observed in all treatment groups. Furthermore, potassium levels after three days at the hospital were also lower compared with the initial values in the control group. Although no significant differences were detected in osmolality values between initial and after fluids administration, the significant lower concentrations of electrolytes detected after fluid therapy could be due to the hypotonic performance of these fluids for sea turtles. The fact that the osmolality observed in turtles from control group showed significant differences between initial and control day reinforces this idea. However, data from previous studies have suggested that terrestrial and marine chelonians can tolerate a wide range of plasma osmolality values without adverse effects [18].

In conclusion, this is the first study evaluating the effects of several crystalloid fluids on the acid-base status and plasma biochemical values in stranded loggerhead sea turtles, providing useful information for the medical care of these protected reptiles. Because metabolic acidosis is the acid-base disorder most frequently detected in stranded sea turtles, we recommend treatment with mixed saline-lactated Ringer’s solution until this acid-base disorder is restored. Furthermore, we provide in this study reference convalescent venous blood gas, acid-base, and plasma biochemical values that may be used as a standard profile, useful for veterinary surgeons involved in sea turtle conservation.

## Supporting Information

S1 Checklist “The ARRIVE Guidelines Checklist” for reporting animal data in this manuscript. (DOCX)
