# Supplementary material for: Acid-Base and Plasma Biochemical Changes Using Crystalloid Fluids in Stranded Juvenile Loggerhead Sea Turtles (Caretta caretta)
Source: PLoS One. 2015 Jul 13;10(7):e0132217. doi: 10.1371/journal.pone.0132217 (PMC4500549; doi:10.1371/journal.pone.0132217)

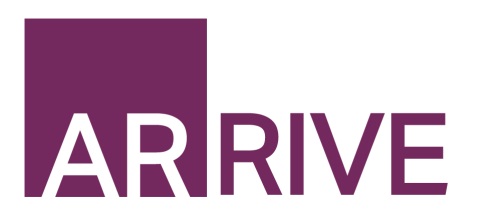


The ARRIVE Guidelines Checklist

Animal Research: Reporting In Vivo Experiments

Carol Kilkenny^1^, William J Browne^2^, Innes C Cuthill^3^, Michael Emerson^4^ and Douglas G Altman^5^

*^1^The National Centre for the Replacement, Refinement and Reduction of Animals in Research, London, UK, ^2^School of Veterinary Science, University of Bristol, Bristol, UK, ^3^School of Biological Sciences, University of Bristol, Bristol, UK, ^4^National Heart and Lung Institute, Imperial College London, UK, ^5^Centre for Statistics in Medicine, University of Oxford, Oxford, UK.*

|  | | ITEM | RECOMMENDATION | Section/ Paragraph |
| --- | --- | --- | --- | --- |
| 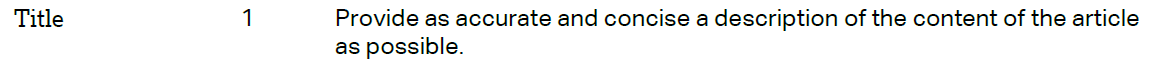 | | | Title |  |
| 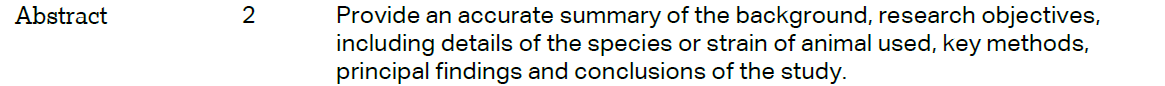 | | | Abstract |  |
| INTRODUCTION | | |  |  |
| 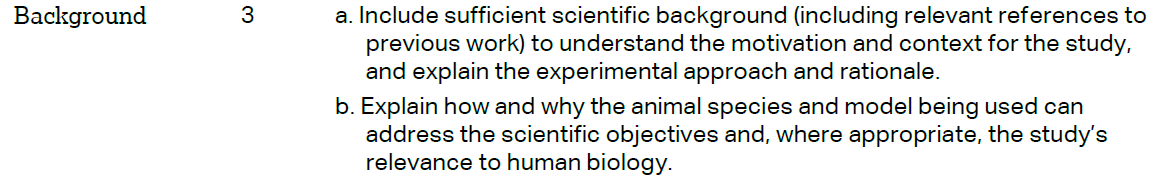 | | | Paragraphs 2-3  Paragraph 3 |  |
| 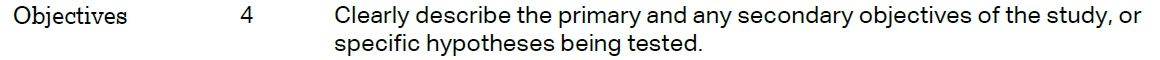 | | | Paragraph 4 |  |
| METHODS | | |  |  |
| 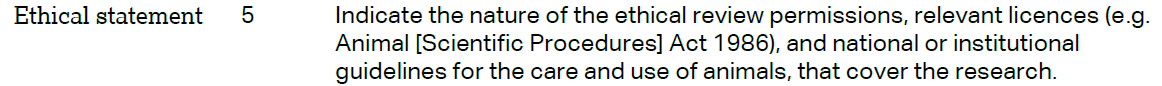 | | | Paragraph 1 (Ethics Statement) |  |
| 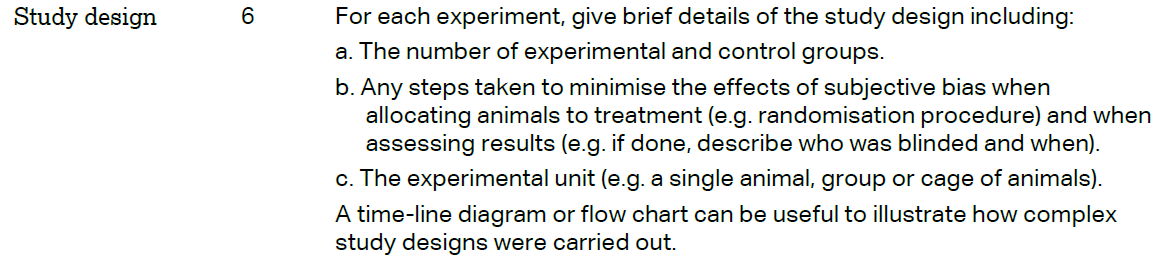 | | | Paragraph 1 (Methodology)  Paragraph 1 (Methodology)  Paragraph 1 (Methodology) |  |
| 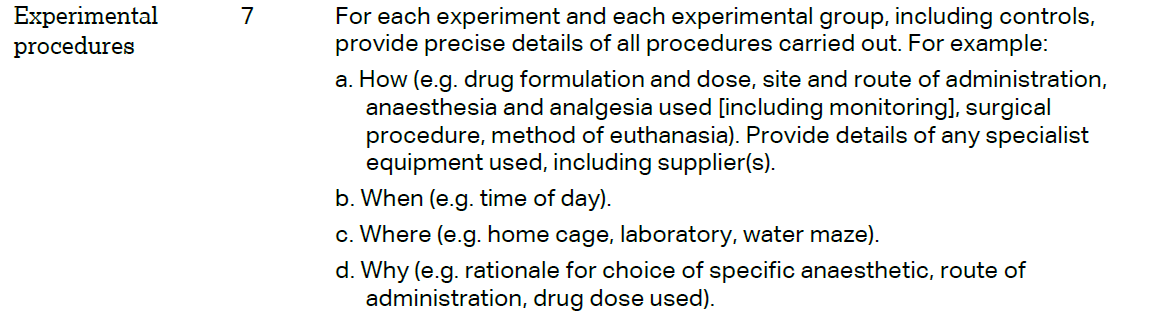 | | | Paragraphs 1-3 (Methodology) |  |
| 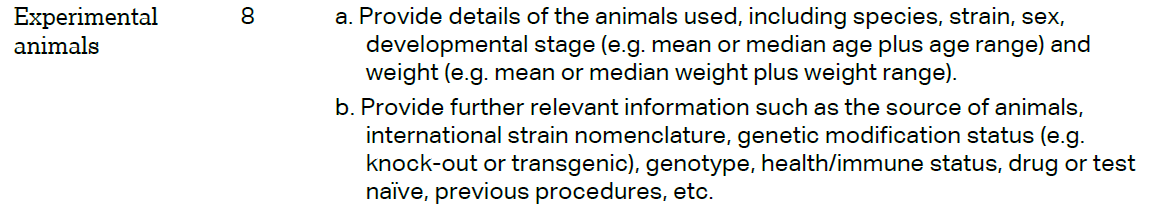 | | | Paragraph 1 (Animals)  Paragraph 1 (Animals) |  |

The ARRIVE guidelines. Originally published in *PLoS Biology*, June 2010^1^

| 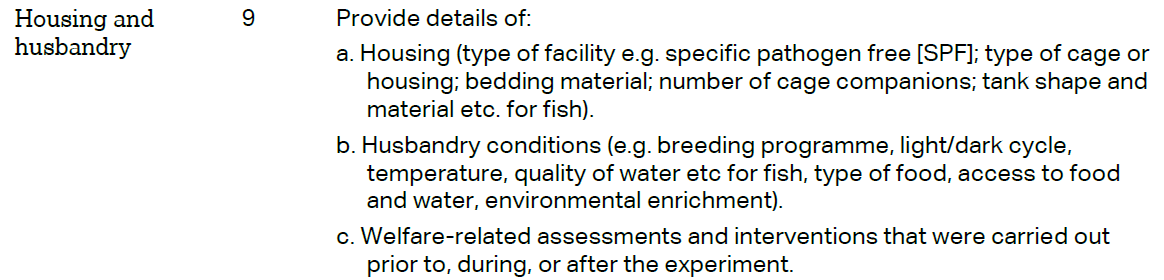 | Paragraph 1 (Methodology)  Paragraph 1 (Methodology)  Paragraph 1 (Methodology) | |
| --- | --- | --- |
| 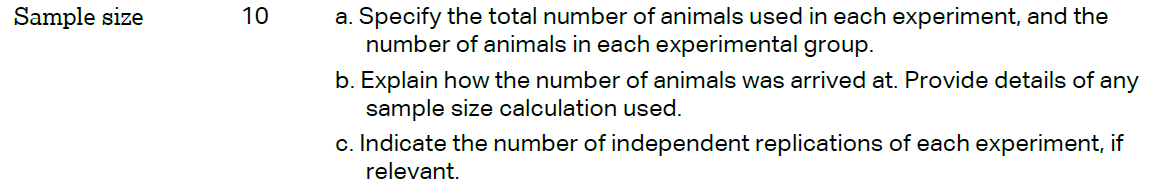 | Paragraph 1 (Animals)  Paragraph 1 (Methodology) | |
| 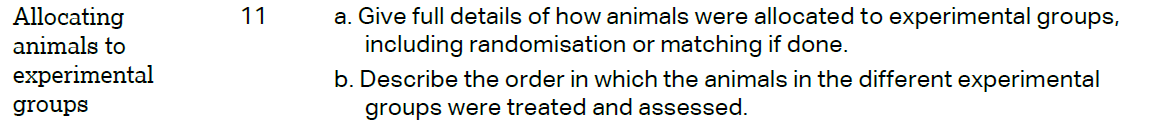 | Paragraph 1 (Methodology) | |
| 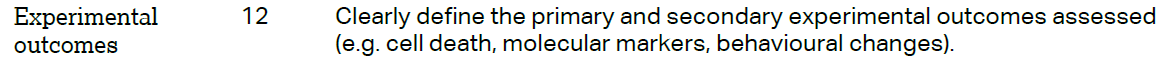 | Paragraph 3 (Methodology) | |
| 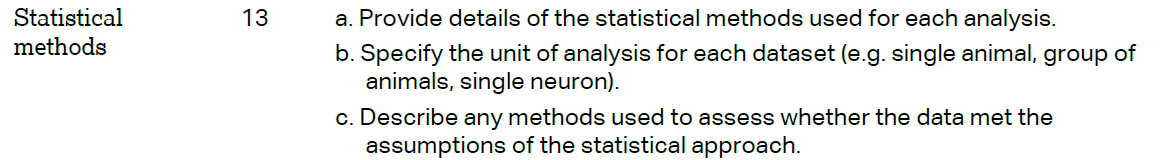 | Paragraph 1 (Statistical analysis) | |
| RESULTS |  | |
| 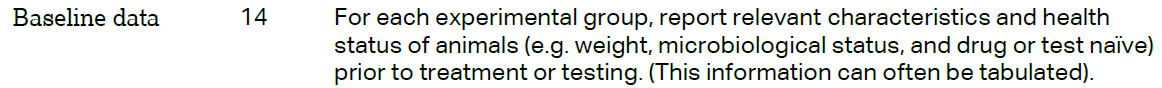 | Tables 1 & 2 (upon admission) | |
| 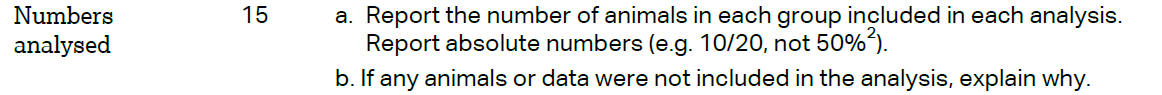 | Paragraph 1 (Methodology) | |
| 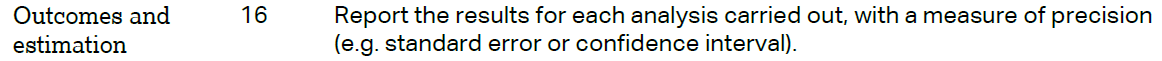 | Tables 1-3 | |
| 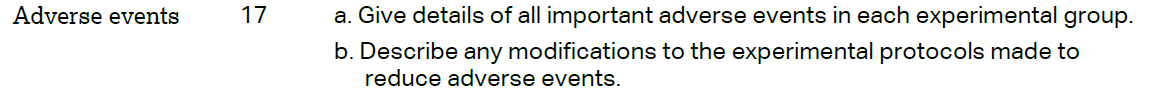 | n.a. | |
| DISCUSSION |  | |
| 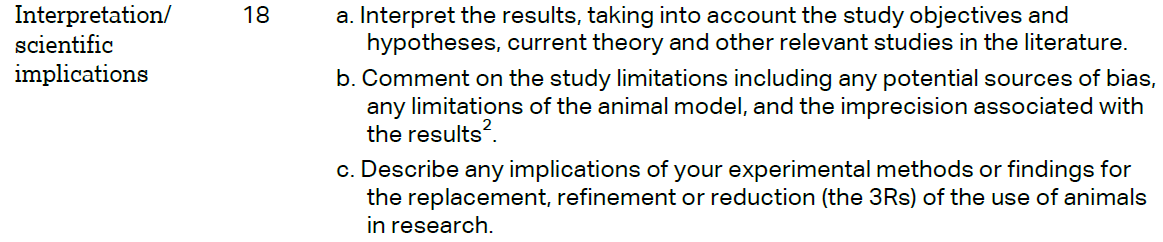 | Throughout Discussion  Paragraph 5 (Discussion) | |
| 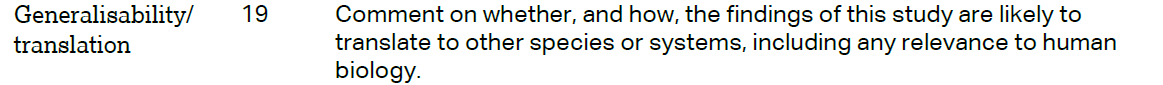 |  | |
| 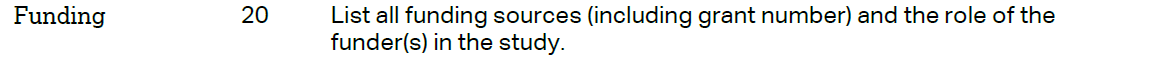 | |  |


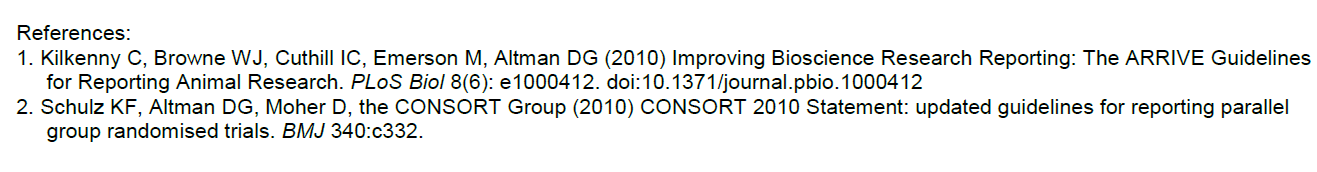

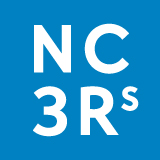

Supplement: S1 Checklist — (DOCX) [file pone.0132217.s001.docx]
